# Design, Synthesis, and In Vitro Evaluation of the Photoactivatable Prodrug of the PARP Inhibitor Talazoparib

**DOI:** 10.3390/molecules25020407

**Published:** 2020-01-18

**Authors:** Jiaguo Li, Dian Xiao, Lianqi Liu, Fei Xie, Wei Li, Wei Sun, Xiaohong Yang, Xinbo Zhou

**Affiliations:** 1 School of Pharmaceutical Sciences, Jilin University, Changchun 130021, China; li_jiaguo@126.com; 2 National Engineering Research Center for the Emergency Drug, Beijing Institute of Pharmacology and Toxicology, Beijing 100850, China; be_xiaodian@163.com (D.X.); llianqi@126.com (L.L.); xiefei0058@163.com (F.X.); a_moon1096@163.com (W.L.)

**Keywords:** Talazoparib, PARP inhibitor, prodrug, o-nitro-benzyl, photoactivatable protecting groups

## Abstract

In this article, we report the design, synthesis, photodynamic properties, and in vitro evaluation of photoactivatable prodrug for the poly (ADP-ribose) polymerase 1 (PARP-1) inhibitor Talazoparib. In order to yield a photoactivatable, inactive prodrug, photoactivatable protecting groups (PPGs) were employed to mask the key pharmacophore of Talazoparib. Our study confirmed the good stability and photolytic effect of prodrugs. A PARP-1 enzyme inhibition assay and PARylation experiment showed that the inhibitory activity of the prodrug was reduced 380 times and more than 658 times, respectively, which proved that the prodrug’s expected activity was lost after PPG protection. In *BRCA1*- and *BRCA2*-deficient cell lines, the inhibitory activity of the compound was significantly restored after ultraviolet (UV) irradiation. The results indicate that the photoactivatable prodrug strategy is an interesting approach for studying PARP inhibitors. Meanwhile, the described photoactivatable prodrug also provided a new biological tool for the mechanism research of PARP.

## 1. Introduction

DNA repair in normal cells mainly relies on the base excision repair (BER) pathway for single-strand DNA breaks and the homologous recombination (HR) pathway for double-strand DNA breaks [1]. Poly (ADP-ribose) polymerase (PARP) plays a key role in the repair of single-strand DNA breaks, through its ability to bind to DNA gaps and recruit other DNA repair enzymes in the BER pathway [1,2,3,4]. The tumor suppressor proteins encoded by the breast cancer genes *BRCA1* and *BRCA2* participate in the HR of double-strand DNA breaks [5]. Usually, in tumor cells with *BRCA1* and *BRCA2* mutations or defects, the inhibition of the PARP enzyme can lead to the accumulation of single-strand DNA breaks, owing to the HR repair pathway being blocked, which causes further double-strand DNA breaks and finally results in the death of tumor cells [6,7,8]. Currently, PARP inhibitors have been a hot topic in the tumor research area. Among them, Olaparib, Rucaparib, Niraparib, and Talazoparib have been approved for the treatment of *BRCA*-deficient breast, ovarian, fallopian tube, and primary peritoneal cancers [9]. In addition, PARP inhibitors are also used in combination with some kinase inhibitors, and show significant synergistic antitumor effects on prostate cancer, pancreatic cancer, gastric cancer, acute leukemia, and non-small-cell lung cancer [10,11,12,13]. The number of diseases illustrate the huge potential for therapeutic agents and for biological probes in PARP research.

Talazoparib (trade name: Talzenna), an oral PARP inhibitor developed by Pfizer, was approved by the U.S. Food and Drug Administration (FDA) on 16 October 2018 [14,15]. It is currently the most active compound among the PARP inhibitors, with an IC_50_ of 0.57 nM, which is 4–10 times lower than that of the other PARP inhibitors [16]. The clinical dose of Talazoparib is only 1 mg (once a day). In contrast, to achieve similar effects as Talazoparib, the dosage of Olaparib [17] and Niraparib [18] are 400 mg (twice a day) and 300 mg (once a day), respectively. In addition, while Talazoparib is highly effective in killing tumor cells, there may still be serious side effects, such as myelodysplastic syndrome or acute myeloid leukemia [14,19]. In light of the immense significance of PARP inhibitors, we aimed to develop a relevant, photoactivatable Talazoparib prodrug.

Photoactivatable prodrugs are usually designed by blocking a key pharmacophore moiety of the inhibitor using photoactivatable protecting groups (PPGs), which have been widely used in biology and medicine in recent years as a non-invasive approach [20,21,22]. This method can provide temporal and spatial control for the release of bioactive substance by UV irradiation. Thus, highly active inhibitors can be generated in irradiated areas of interest at defined points in time. The photoactivatable prodrugs can serve as a novel biological tool for kinetic or mechanistic studies. Moreover, blocked inhibitors may minimize the systemic side effects of Talazoparib, in order to enable a higher dosage of inactive prodrugs.

**Figure 1:**
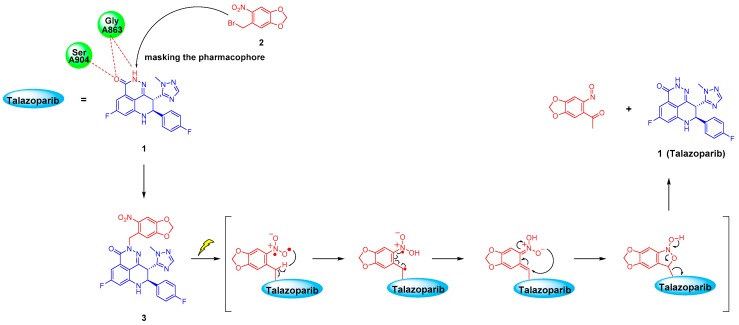
Photolysis mechanism of o-nitrobenzyl upon 365 nm UV irradiation and the inferred drug release process of the Talazoparib prodrug.

The o-nitrobenzyl system and its derivatives are one of the most commonly used PPGs [23,24,25,26]. The photolysis mechanism is intramolecular rearrangement. As shown in Figure 1, under 365 nm UV irradiation, carbonyl compounds containing o-nitrobenzyl can form a highly active bi-radical, which will undergo hydrogen abstraction on the carbon atom at the γ position and release the parent drugs [26,27,28,29]. The advantages of such PPG technology are a high release speed and easy chemical synthesis. At the same time, the UV release wavelength of 365 nm involves less DNA damage than wavelengths shorter than 300 nm, and is harmless to tissues and cells at low doses [29,30]. This method has potential clinical application value [25,26]. For example, irradiation can be temporarily applied to the lesion site during or after surgery to release active substances for killing residual cancer cells, thereby preventing postoperative recurrence. Optical fibres and endoscopic probes can also be used to transmit the required light to the action site to treat some superficial tumors.

In this study, we report the design, synthesis, photodynamic properties, and in vitro evaluation of a photoactivatable prodrug for the PARP-1 inhibitor Talazoparib. We used the concept of PPGs to covalently bond o-nitrobenzyl derivatives (herein designated as compound **2**) to the lactam pharmacophore of Talazoparib, under the guidance of molecular docking; this disrupts its key hydrogen bonding interaction with the PARP protein residue to yield an inactive photoactivatable prodrug, i.e., compound **3**. Our research proved that newly designed prodrug **3** showed good stability and could be rapidly deprotected after UV radiation. A PARP-1 enzyme inhibition assay and PARylation experiment showed that inhibitory activity of the PPG-protected prodrug was greatly reduced by 380 and more than 658 times, respectively. In addition, in *BRCA1*- and *BRCA2*-deficient cell lines, the inhibitory activity of the prodrug **3** was significantly restored after transitory UV irradiation, and the data implies that inhibitory activity could increase with prolonged irradiation time. The results of this preliminary study indicate that the photoactivatable prodrug strategy was an interesting approach for studying PARP inhibitors. Meanwhile, the described photoactivatable prodrugs also provide a novel biological tool for the signal transduction research.

## 2. Results and Discussion

### 2.1. Molecular Modelling

**Figure 2:**
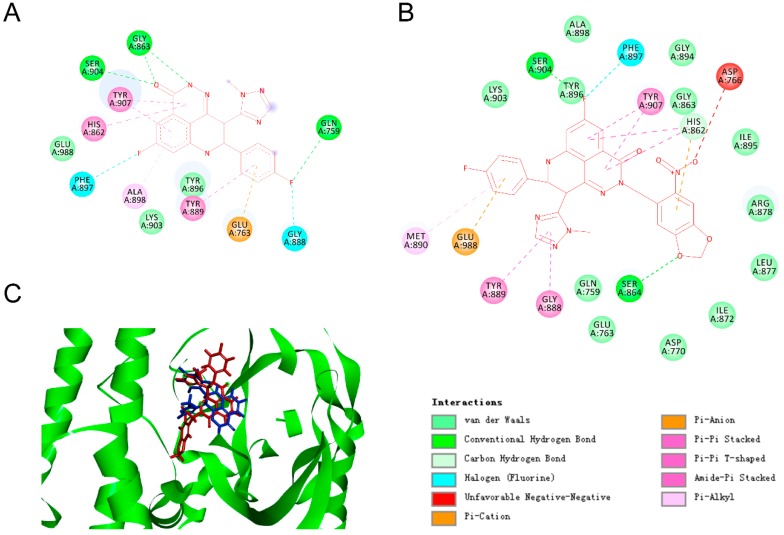
Predicted ligand binding models relative to the catalytic domain of poly (ADP-ribose) polymerase 1 (catPARP-1). (**A**) The co-crystallized binding modes of Talazoparib and the catPARP-1 protein were taken from PDB ID 4PJT. (**B**) Predicted co-crystallized binding poses of compound **3** in a complex with catPARP-1. (**C**) Superimposition of the binding models of Tazaloparib (blue) and compound **3** (red) with catPARP-1 protein (green).

Molecular docking of the Tazaloparib into the catalytic domain of PARP-1 (catPARP-1) revealed that its lactam moiety was the key pharmacodynamic group (PDB ID 4PJT). N and O atoms on the lactam moiety could form hydrogen bonds with the Gly863 and Ser904 residues of PARP-1, respectively (Figure 2A) [31,32]. To block its pharmacophoric features, we linked PPGs (namely, compound **2**) to the lactam moiety to form compound **3**, and simulated the action mode of this compound in the same activity pocket. Consistent with our hypothesis, no proper binding of compound **3** to the enzyme active sites were found during the docking; that is, the PPGs blocked the interaction of the lactam moiety with the Gly863 and Ser904 residues (Figure 2B). In addition, the introduction of PPGs led to spatial conflict, which rendered compound **3** unable to maintain the same binding mode as Talazoparib, and the whole molecule was turned over by a certain angle degree (Figure 2C). Inspired by this docking result, we synthesised compound **3** and carried out the following series of photochemical characterisations and biological evaluations.

### 2.2. Chemistry

**Scheme 1:**
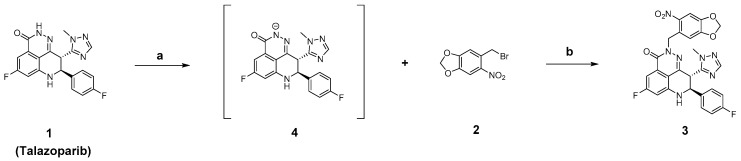
Synthesis of compound **3**. Reagents: (a) n-BuLi, tetrahydrofuran (THF), N_2_, –80 °C, 10 min; (b) THF, N_2_, 0 °C, 1 h.

As shown in Scheme 1, Talazoparib could generate the intermediate compound **4** under the action of n-Butyllithium. Compound **4** subsequently underwent a nucleophilic substitution reaction with compound **2** to generate the photoactivatable target compound **3**.

### 2.3. Stability Assays and UV Cleavage Test In Vitro

#### 2.3.1. UV Stability of Talazoparib

**Figure 3:**
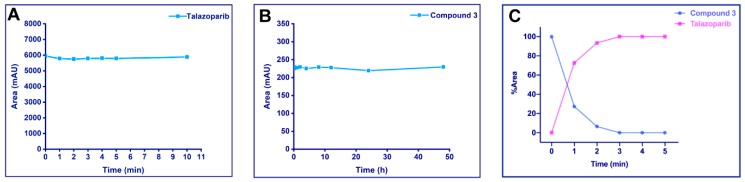
Stability assays and UV cleavage test in vitro. Compounds were irradiated at 365 nm (4.1 mW/cm^2^). Samples were determined on a C18 column using a water/acetonitrile (ACN) gradient. The detection wavelength for high-performance liquid chromatography (HPLC) analysis was 220 nm. The peak area or peak area percentage was plotted against the irradiation time. (**A**) UV stability of Talazoparib: 2.63 mM Talazoparib in methanol were irradiated up to 10 min, and aliquot samples at different time points were analyzed by HPLC. Talazoparib was stable under the described UV irradiation. (**B**) Phosphate-buffered saline (PBS) stability of the photoactivatable prodrug: 20 μM compound **3** in pH = 7.4 PBS (containing 10% DMSO) were incubated at 37 °C for 48 h and analyzed by HPLC. The peak area of compound **3** did not show significant changes. (**C**) UV cleavage test on compound **3**: 20 μM of the compound in methanol was irradiated at 365 nm for 5 min and analyzed by HPLC. By progressing irradiation, compound **3** was converted into Talazoparib.

Talazoparib should have sufficient stability at the light radiation wavelength used; otherwise, it will be degraded immediately after release or even before the breaking of the PPG covalent bond. Therefore, we first evaluated the UV stability of Talazoparib at a wavelength of 365 nm. To this end, we used a UV cross-linker with an emission wavelength of 365 nm (4.1 mWs/cm^2^) to irradiate 2.63 mM Talazoparib in methanol, and took aliquots of the sample at different times for high-performance liquid chromatography (HPLC) analysis. The result indicated that there was no significant loss of Talazoparib in the peak area within 10 min, showing good UV stability (Figure 3A). In addition, the UV/vis absorption spectra of Talazoparib and compound **3** was shown in supplementary materials (See Figure S1).

#### 2.3.2. Stability of the Photoactivatable Prodrug in Phosphate-Buffered Saline

The synthesised photoactivatable prodrug should be sufficiently stable. If a high dose of inactive prodrugs were converted to the parent drug in advance, this might produce some undesirable toxic and side effects. Therefore, we tested the stability of compound **3** in phosphate-buffered saline (PBS) with the same pH as blood. Then 20 μM of compound **3** were added to pH = 7.4 PBS containing 10% dimethyl sulfoxide, which was incubated in a 37 °C thermostatic incubator for 48 h under normal brightness, during which samples of equal concentration were taken at different time points for HPLC analysis. The results were plotted according to the sampling time points and the peak areas of the compound (Figure 3B). The peak area of compound **3** did not show significant changes within 48 h, indicating that the compound **3** had good stability in PBS.

#### 2.3.3. UV Release of the Photoactivatable Prodrug

A photoactivatable prodrug needs to not only have good stability, but also be rapidly and effectively released at the irradiated site. Therefore, we tested the photolysis of compound **3**. Figure 3C shows the release situation of compound **3** (20 μM in methanol) after UV irradiation. Under the current experimental conditions, compound **3** could rapidly and completely release Talazoparib in 3 min. In addition, the release experiment also provided a reference for the irradiation duration to be used for the subsequent cell experiments.

### 2.4. Enzymatic Experiments In Vitro

#### 2.4.1. Inhibition of the PARP-1 Enzyme

**Figure 4:**
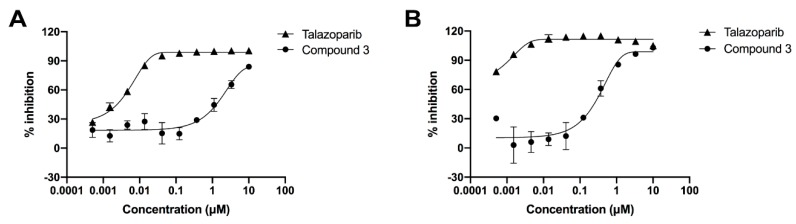
(**A**) Dose–response curve of Talazoparib and compound **3** on PARP-1 enzyme inhibition. Compound **3** showed a significant decrease in the inhibitory activity compared to the active Talazoparib to PARP-1 enzyme. Error bars represent standard deviation from duplicate determinations, and results are shown as mean ± standard deviation (SD). The IC_50_ value is presented in Table 1. (**B**) Dose–response curve of Talazoparib and compound **3** on PARylation assay. The ability of compound **3** to inhibit the polymerization of PAR significant decreased compared to the active Talazoparib in Hela cells. Error bars represented standard deviation from duplicate determinations, and results are shown as mean ± SD. The IC_50_ value is presented in Table 2.

According to the guidance of molecular docking, after the PPG was introduced at the lactam pharmacophore of Talazoparib, the prodrug **3** should have had a reduced inhibitory effect on the PARP-1 enzyme. Therefore, we evaluated the inhibitory activities of Talazoparib and compound **3** on PARP-1. As shown in the dose-dependent curve in Figure 4A, the inhibitory activity of the PPG-inactivated compound **3** was significantly lower than that of Talazoparib. The IC_50_ values of Talazoparib and compound **3** were 0.005 and 1.919 μM, respectively, indicating that the inhibitory activity was reduced by 380 times (Table 1). This was consistent with the prediction from the molecular docking. The test results strongly proved that the interaction between the inhibitor and PARP-1 enzyme could be significantly blocked after introducing PPG to the lactam pharmacophore of Talazoparib.

#### 2.4.2. PARylation Assay

The inhibitory activity of compounds on the PARP-1 enzyme could also be evaluated at the cellular level by using a hydrogen peroxide solution to damage the DNA of cells. Under normal circumstances, PARP-1 would be activated immediately, and the poly (ADP-ribose) polymer would be synthesised with NAD+ as the substrate. In the presence of a PARP-1 inhibitor, however, the biological function of PARP-1 would be inhibited. Therefore, the inhibitory activity on PARP-1 can be determined by detecting the level of poly (ADP-ribose) [16,33]. From the data in Figure 4B and Table 2, it can be seen that Talazoparib showed a strong inhibitory effect on poly ADP-ribosylation in HeLa cells, with an IC_50_ value of less than 0.0005 μM, whereas compound **3** showed a weak inhibitory effect, with an IC_50_ value of 0.329 μM, a decrease of 658 times at least.

### 2.5. Compound’s Cytotoxicity Assay in the Absence and Presence of UV Irradiation

**Figure 5:**
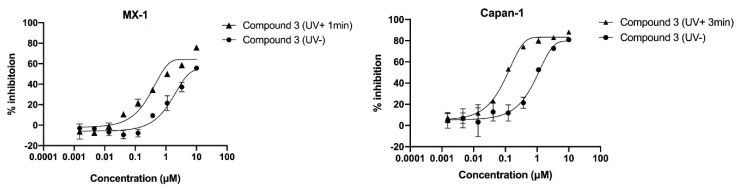
Dose–response curves of compound **3** in the absence and presence of ultraviolet irradiation. Cells were incubated for 1 h with the compounds and were then irradiated at 365 nm (4.1 mW/cm^2^). Cell growth was determined 10 days after incubation with the compounds. Irradiated compounds showed left-shifted dose–response curve compared to that of the unirradiated compounds. Error bars represent the standard deviation from duplicate determinations, and results are shown as mean ± SD. The IC_50_ value is presented in Table 3.

After confirming that the introduction of PPG could block the inhibitory activity of Talazoparib on PARP-1, we speculated that compound **3** should also have less cytotoxicity than Talazoparib. To verify this idea, cell proliferation inhibition experiments were conducted. As Talazoparib should only be applied to *BRCA1* or BRCA*2*-defective cell lines, the principle is simultaneous inhibition of the BER pathway and HR repair pathway of DNA to produce a synthetic lethality effect and consequently, the apoptosis of the tumour cells. Therefore, we selected the well-recognized *BRCA1*- and *BRCA2*-defective cell lines MX-1 and Capan-1 for the following tests. The results indicate that Talazoparib exhibited effective cytotoxicity in the MX-1 and Capan-1 cell lines (IC_50_ values: 0.015 μM and 0.003 μM, respectively). In contrast, the PPG-inactivated compound **3** showed no significant cytotoxicity, with IC_50_ values of 1.873 μM and 0.863 μM, respectively (Figure 5 and Table 3).

**Figure 6:**
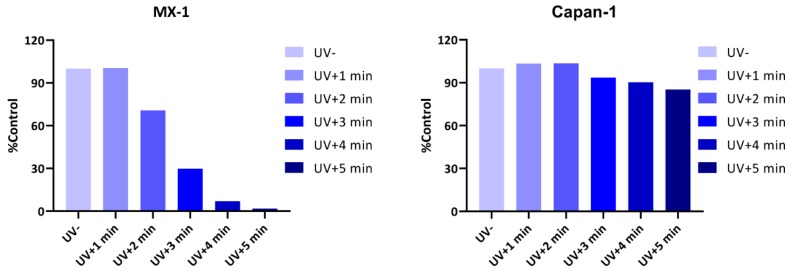
Cellular ultraviolet light tolerance test. Cells were irradiated at 365 nm (4.1 mW/cm^2^) for indicated periods of time, and cell growth was determined after 10 days. The MX-1 cell line was relatively sensitive to UV irradiation. Radiation over 1 min affected cell proliferation. In contrast, the Capan-1 cell line tolerated UV irradiation for a relatively long time.

Next, we tested whether the inhibitory activity of compound **3** could be restored after UV irradiation. In order to negate the influence of UV irradiation on cells, to avoid interfering with the experiment results, we first measured the maximum tolerance of cells to UV light. As shown in Figure 6, the MX-1 cell line was relatively sensitive to UV irradiation, and could tolerate an irradiation duration of 1 min, whereas further extension of the duration would affect cell proliferation. In contrast, the Capan-1 cell line could tolerate UV irradiation for a relatively long time, and approximately 85% of the cells were still alive after irradiation for 5 min. On the basis of the principle of maximising drug release, while ensuring normal cell proliferation as much as possible, we determined the UV irradiation durations for the MX-1 and Capan-1 cells to be 1 min and 3 min, respectively.

As shown in Table 3, the inhibitory activity of compound **3** on MX-1 cells after UV irradiation was approximately three times higher than that on the non-irradiated group (IC_50_ values: 1.873 μM vs. 0.577 μM, respectively). However, it did not reach the same level of inhibition as the parent drug Talazoparib, likely because the irradiation duration of 1 min was not enough to allow the release of all the compound **3**. Therefore, we tested the Capan-1 cells that could tolerate a longer UV irradiation duration. To our delight, the IC_50_ value of compound ***3*** for Capan-1 cells was 0.092 μM after 3 min of irradiation, which was nine times higher than that of the non-irradiated group (IC_50_ value: 0.863 μM). Further analysis was needed to determine the reason why the inhibitory activity of compound **3** still did not reach the same level as Talazoparib after UV irradiation. We simulated the same cytotoxicity experimental conditions to evaluate the photolysis of 10 μΜ compound **3** (10 μM is the starting concentration of compound **3** in cytotoxicity experiments) in a 96-well plate. The results showed that compound **3** was not released completely after 3 min UV irradiation (see Figure S2). 

The above cytotoxicity experiment data indicated that compound **3** could significantly restore the effective activity of Talazoparib after being irradiated by UV light, and the data from both cells imply that inhibitory activity could increase with prolonged irradiation time. However, owing to the limited types of *BRCA*-deficient cells that could be selected for study and their restrictive UV light tolerance, compound **3** did not reach the same level of inhibitory activity as Talazoparib. The experimental results could provide inspiration for future research. 

### 2.6. Verification of the Toxicity of Leaving Photoactivatable Protecting Groups

**Scheme 2:**
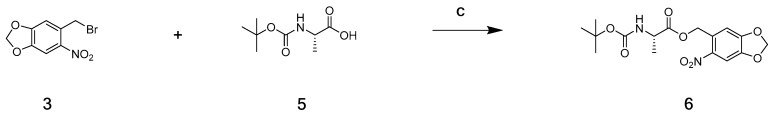
Synthesis of compound **6**. Reagents: (c) K_2_CO_3_, reflux, 5 h.

Subsequently, the leaving PPG after irradiation was also evaluated for its cytotoxicity. Compound **3** was obviously not suitable for verifying this, since it released active Talazoparib after its photocleavage. Therefore, we used an indirect test method by synthesising compound **6** via the nucleophilic substitution reaction between the non-cytotoxic Boc-protected amino acid L-alanine (Boc-Ala), as reported in the literature, and compound **2** under the catalysis of potassium carbonate (Scheme 2), and examined compound **6′**s effect on cell proliferation before and after irradiation [25,26].

**Figure 7:**
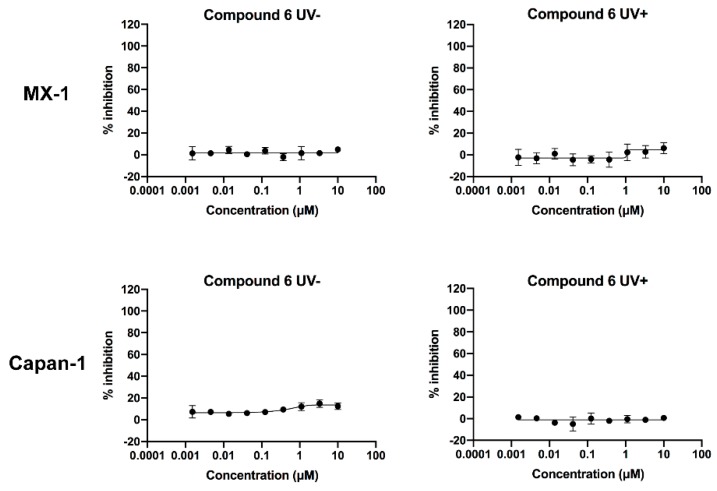
Dose–response curves of compound **6** in the absence and presence of ultraviolet radiation in the MX-1 and Capan-1 cells. After 20 min of irradiation at 365 nm (4.1 mW/cm^2^), the compounds were transferred to 96-well plates and incubated with the cells. Cell growth was determined 10 days after incubation with the compounds. Irradiated compounds showed similar dose–response curves as that of unirradiated compounds. Error bars represent standard deviation from duplicate determinations, and results are shown as mean ± SD. The IC_50_ value is presented in Table 4.

We first evaluated the photolysis of compound **6**, and the experimental results showed that 20 μM of compound **6** in methanol solution were almost completely released within 20 min (see Figure S3). Next, we used the same initial concentration as that of compound **3** for the measurement. To fully release the drug, we UV-irradiated compound **6** at different concentrations for 20 min, and then added each sample to cells for co-culture. As a result, compound **6** showed no significant cytotoxicity in MX-1 and Capan-1 cells both before and after irradiation (Figure 7 and Table 4). Since the Boc–Ala released after irradiation was not cytotoxic, the results verified that the leaving PPG at the indicated concentrations did not cause any cytotoxicity. This therefore proved that the cytotoxicity of compound **3** after irradiation could be attributed entirely to the released Talazoparib.

## 3. Materials and Methods 

### 3.1. Molecular Docking

The catPARP-1 crystal structure (PDB ID 4PJT) was downloaded from the Protein Data Bank. Molecular docking was carried out using Gold (The Cambridge Crystallographic Data Centre). Before docking calculations, water molecules of the crystal structure were deleted, and hydrogen atoms were added. The whole 4PJT was defined as a receptor, and the site sphere was selected based on the active site of 4PJT. Compound **3** was placed during the molecular docking procedure. After the end of molecular modelling, types of interactions of the docked protein with ligand were analyzed.

### 3.2. Chemistry

All of the reagents and solvents were purchased from Innochem (Beijing, China), Aladdin (Shanghai, China), Energy Chemical (Shanghai, China), TCI (Tokyo, Japan), Ark Pharm (Libertyville, IL, USA), and used without additional purification. Anhydrous solvents were stored in sure-seal bottles under dry nitrogen. Analytical thin-layer chromatography was conducted on pre-coated silica gel plates (Yantai Dexin Biotechnology Co., Ltd., Yantai, China). Visualization was accomplished with 254 nm and 365 nm UV light. Column chromatography was performed using silica gel (200–300 mesh; Qingdao Ocean Chemical Engineering Co., Ltd., Qingdao, China). 

Nuclear magnetic resonance (NMR) spectra were obtained on a JNM-ECA-400 400 MHz spectrometer (JEOL Ltd., Tokyo, Japan). Chemical shifts were reported in ppm and TMS was used as the internal standard. Coupling constants (*J*) are given in Hertz. Spin multiplicities were reported as the following abbreviations: s (singulet), d (doublet), dd (doublet doublet), t (triplet), q (quadruplet), m (multiplet). The mass spectrometry (MS) systems were the API 3000 triple-quadrupole mass spectrometer equipped with a Turbo Ion Spray electrospray ionization (ESI) source (AB Sciex, Concord, ON, Canada). HPLC analysis was performed using an Agilent 1260 Series (California, CA, USA).

#### 3.2.1. Synthesis of (8*S*,9*R*)-5-Fluoro-8-(4-fluorophenyl)-9-(1-methyl-1*H*-1,2,4-triazol-5-yl)-2-((6-nitrobenzo[d][1,3]dioxol-5-yl)methyl)-2,7,8,9-tetrahydro-3*H* pyrido[4,3,2-de]phthalazin-3-one (**3**)

To a solution of Talazoparib (70 mg, 0.18 mmol) in anhydrous tetrahydrofuran (THF) (30 mL) was added to a 100 mL, two-necked flask with a magnetic stir bar. The flask was evacuated and backfilled with argon three times. Then 2.5 M n-Butyllithium (108 μL, 0.27 mmol) in hexanes were added the reaction solution, and the mixture was cooled at −80 °C. After stirring for 10 min, a solution of 5-(bromomethyl)-6-nitrobenzo[d][1,3] dioxole (93.9 mg, 0.27 mmol) in anhydrous THF was slowly added to the mixture, was warmed to 0 °C, and stirred overnight. When the reaction was completed, the mixture was carefully quenched by ammonium chloride saturated solution, and was extracted with ethyl acetate (50 mL × 3). The combined organic layers were washed with brine, dried over anhydrous sodium sulfate, and concentrated. The crude product was purified by column chromatography (dichloromethane/methanol = 50:1) to give compound **3** (46 mg, yield 45.7%) as a light yellow solid; ^1^H NMR (400 MHz, DMSO-*d*_6_) δ 7.79 (s, 1H); 7.73 (s, 1H); 7.63 (s, 1H); 7.53–7.45 (m, 2H); 7.20–7.13 (m, 2H); 7.10 (dd, *J* = 9.0, 2.4 Hz, 1H); 6.93 (dd, *J* = 11.1, 2.5 Hz, 1H); 6.61 (s, 1H); 6.22–6.21 (m, 2H); 5.40–5.29 (m, 2H); 5.08–5.00 (m, 2H); 3.56 (s, 3H). ESI m/z (M + H)^+^ calculated for C_27_H_20_F_2_N_7_O_5_^+^ 560.15 found 560.15.

#### 3.2.2. (6-Nitrobenzo[d][1,3]dioxol-5-yl)methyl (tert-butoxycarbonyl)-l-alaninate (**6**)

To a solution of (tert-butoxycarbonyl)-l-alanine (190 mg, 1.0 mmol) in acetone, potassium carbonate (207.31 mg, 1.50 mmol) and compound **3** were added. Then the mixture was stirred and heated at reflux for 5 h. After the reaction completed, the mixture was cooled to room temperature and removed under reduced pressure. The residue was dissolved in water and then the aqueous solution was extracted with dichloromethane (50 mL × 3). The combined organic layers were washed with brine, dried over anhydrous sodium sulfate, and concentrated to give the crude product, which was purified by column chromatography (ethyl acetate/petroleum ether = 5:1) to give compound **6** (264 mg, yield 71.4%) as a light yellow solid; ^1^H NMR (400 MHz, DMSO-*d*_6_) δ 7.74 (s, 1H); 7.46 (d, *J* = 7.3 Hz, 1H); 7.22 (s, 1H), 6.26 (s, 2H); 5.43–5.32 (m, 2H); 4.15–4.08 (m, 1H); 1.38 (s, 9H); 1.27 (d, *J* = 7.4 Hz, 3H). ESI m/z (M + Na)^+^ calculated for C_16_H_20_N_2_NaO_8_^+^ 391.11 found 391.11.

### 3.3. Talazoparib UV Stability Assays

Talazoparib was dissolved in methanol (2.63 mM), and the solution was divided into six equal portions for UV irradiation at 365 nm by using an UV crosslinker (CL-1000L UVP Crosslinker, 5 × 8 W; Analytikjena, IL, USA). The irradiation times were 1 min, 2 min, 3 min, 4 min, 5 min, and 10 min. The irradiated solution was transferred to a volumetric flask and diluted to the same volume with methanol. Then HPLC analysis was performed to detect the compound peak area. Samples were determined on an Agilent Eclipse Plus C18 column (4.6 × 150 mm, 5 μm particle size; California, CA, USA) using a mobile phase A (water) and mobile phase B (acetonitrile (ACN)) to elute. The gradient changed from 70% A to 30% A in 10 min, then continuing to elute with 30% A for 5 min. The flow rate was 1 mL/min, and the detection wavelength was 220 nm.

### 3.4. Phosphate-Buffered Saline Stability of the Photoactivatable Prodrug

Twenty μM of the test compound in pH = 7.4 PBS, containing 10% DMSO, were transferred to 13 1 mL volumetric flasks in equal volumes, incubated at 37 °C. The volumetric flasks were taken out at 0 min, 5 min, 10 min, 15 min, 30 min, 1 h, 2 h, 4 h, 8 h, 12 h, 24 h, 48 h, and 7 days, and were frozen at −80 °C. The samples were thawed in sequence and diluted to the same volume with methanol. Then HPLC analysis was performed to detect the compound peak area. Samples were determined on an Agilent Eclipse Plus C18 column (4.6 × 150 mm, 5 μm particle size; California, CA, USA) using a mobile phase A (water) and mobile phase B (ACN) to elute. The gradient changed from 70% A to 30% A in 10 min, continuing to elute with 30% A for 5 min. The flow rate was 1 mL/min, and the detection wavelength was 220 nm.

### 3.5. UV Cleavage Test for Photoactivatable Prodrug

The compound was dissolved in methanol (20 μM), and the solution were irradiated at 365 nm by using an UV crosslinker. Every minute from 0 to 10 min, aliquot samples (200 μL) were determined on an Agilent Eclipse Plus C18 column (4.6 × 150 mm, 5 μm particle size) using mobile phase A (water) and mobile phase B (ACN) to elute. The gradient changed from 70% A to 30% A in 10 min, continuing to elute with 30% A for 5 min. The flow rate was 1 mL/min and the detection wavelength was 220 nm. The peak areas percentage of the compounds at each time point were plotted against the sampling time.

### 3.6. PARP-1 Enzyme Inhibition Assay

A PARP-1 Chemiluminescent Assay Kit (Cat# 80569, BPS, San Diego, CA, USA) was used to detect the ability of compounds to inhibit PARP-1 enzyme activity. All experimental steps followed the operating manual. Firstly, each well was precoated with 25 μL 1× histone mixture that was diluted in PBS by incubation at 4 °C overnight. The wells were blocked by adding 100 μL of blocking buffer, then incubated at room temperature for 90 min. Then 12.5 μL of the prepared 1× PARP assay mixture and 1× activated DNA in 1× PARP buffer were added into each well of the assay plate, and 2.5 μL of compound or solvent control were added at varying concentrations. The reaction was initiated by the addition of 10 μL 1 ng/μL PARP-1 enzyme in 1× PARP buffer at room temperature for 60 min. Next, the reaction solution was added with 25 μL of diluted Streptavidin-HRP (1:50 in blocking buffer) to each well at room temperature for an additional 30 min. Finally, 25 μL of Horseradish Peroxidase (HRP) chemiluminescent substrate A and 25 μL of HRP chemiluminescent substrate B were mixed on ice, and the mixture was added by 50 μL per well. The luminescent signal was measured using a multi-function microplate reader, and IC_50_ values were calculated using GraphPad Prism 8.0 software.

### 3.7. PARylation Assay

The cellular PARylation assay evaluated the ability of test compounds to inhibit polymerization of PAR. Hela cells were seeded in 96-well plates (10,000 cells/well) in 200 μL of cell complete medium, and were allowed to adhere for 4 h at 37 °C in a humidified atmosphere of 5% CO_2_. The medium was then removed from the plates, and the cells were treated with varying concentrations of test compounds for 18 h. 100 μL serum-free medium with 500 μM H_2_O_2_ solution was added, and the plate was kept at 37 °C for 5 min. Next, cells were fixed for 20 min with prechilled methanol at −20 °C and were incubated with 60 μL Poly(ADP-ribose) monoclonal antibody (1:2000) (prepared at 1x PBS containing 0.05% Tween-20 with 1% bovine serum albumin) at 37 °C for 2 h, followed by incubation with 60 μL IRDye 800CW goat anti-mouse IgG (diluted to 1:5000) and DNA stain DRAQ5 (1:5000) (prepared at 1× PBS containing 0.05% Tween-20 with 1% bovine serum albumin) at 37 °C for 2 h. The fluorescent signal was normalized with the DRAQ5 signal, and IC_50_ values were calculated using GraphPad Prism 8.0 software.

### 3.8. Cell Proliferation Assay

To assess the cytotoxicity of test compounds on *BRCA*-deficient cells using a CellTiter-Glo assay. MX-1 cells (*BRCA1*-deficient) and Capan-1 cells (*BRCA2*-deficient) were seeded in 96-well plates at densities that allowed linear growth for 10 days, and adhered overnight at 37 °C in a humidified atmosphere of 5% CO_2_. Cells were treated in their recommended growth medium containing varying concentrations of test compounds. When the cells were incubated with the test compounds for 1 h, the cell culture plates were irradiated to the corresponding time with an ultraviolet crosslinker (for compounds that did not require UV irradiation, this step could be omitted). After 10 days of incubation, CellTiter-Glo Reagent (Promega, Madison, WI, USA) was added to the plates, which continued to be incubated for 30 min at room temperature. Chemiluminescence values were recorded by a multiplate reader, and IC_50_ values were calculated using GraphPad Prism 8.0 software.

## 4. Conclusions

In summary, this paper reports, for the first time, a photoactivatable Talazoparib prodrug. During the research process, we blocked the key lactam pharmacophore of Talazoparib with PPGs under the guidance of computer molecular docking. The in vitro enzyme inhibition assay and PARylation experiment proved that the inhibitory activity of the PPG-inactivated compound was greatly reduced (by 380 and more than 658 times, respectively). In *BRCA1*- and *BRCA2*- deficient cell lines, the prodrug could significantly restore the effective activity of Talazoparib after being irradiated by UV light, and the data imply that inhibitory activity could increase with prolonged irradiation time. However, because of the limitations of the relatively few types of *BRCA*-deficient cells that could be selected for study, as well as their restricted tolerance to UV light, we could not obtain the same level of inhibitory activity from the irradiated prodrug as from the parent drug. Further structural optimisation of the compound and appropriate cell selection would be helpful for further research on this photoactivatable Talazoparib prodrug strategy.

In conclusion, the results of this preliminary study prove that the photoactivatable prodrug strategy was an interesting approach for studying PARP inhibitors. On the one hand, the described photoactivatable prodrug could provide a new biological tool for the mechanism research of PARP. On the other hand, photoactivatable prodrug also create new possibilities for therapeutic applications. However, this requires profound research on the prodrug’ stability, toxicity, and bioavailability, and further cell and animal studies will be planned to address these questions.

## Figures and Tables

**Table 1:** Poly (ADP-ribose) polymerase 1 (PARP-1) enzyme inhibitory activity of Talazoparib and compound **3**.

Test Compound	Inhibition of PARP-1 Enzyme
	IC_50_ (μM)
Talazoparib	0.005
**3**	1.919

**Table 2:** Inhibitory activity of Talazoparib and compound **3** on poly (ADP-ribose) (PAR) polymerization.

Test Compound	Inhibit Polymerization of PAR
	IC_50_ (μM)
Talazoparib	<0.0005
**3**	0.329

**Table 3:** Capan-1 and MX-1 cell inhibitory activities of Talazoparib and compound **3** in the absence and presence ultraviolet radiation.

Test Compound	MX-1 Cell (UV + 1 min) IC_50_ (μM)	Capan-1 Cell (UV + 3 min IC_50_ (μM)
Talazoparib (UV-)	0.015	0.003
**3** (UV-)	1.873	0.863
**3** (UV+)	0.577	0.092

**Table 4:** MX-1 and Capan-1 cell inhibitory activities of compound **6** in the absence and presence ultraviolet radiation.

Test Compound	MX-1 Cell IC_50_ (μM)	Capan-1 Cell IC_50_ (μM)
**6** (UV-)	>10	>10
**6** (UV+)	>10	>10

## Supplementary Materials

The following are available online at https://www.mdpi.com/1420-3049/25/2/407/s1, Figure S1: UV/Vis absorption spectra of compound 3 and Talazoparib in methanol solution, Figure S2: UV-cleavage test on the compound 3 in culture medium DMEM, Figure S3: UV-cleavage test on the compound 6, Figure S4 and S6: The ^1^H-NMR spectrometry of compound 3 and 6, Figure S5 and S7: Mass spectrometry of compound 3 and 6.
