# Supplementary material for: Design, Synthesis, and In Vitro Evaluation of the Photoactivatable Prodrug of the PARP Inhibitor Talazoparib
Source: Molecules. 2020 Jan 18;25(2):407. doi: 10.3390/molecules25020407 (PMC7024556; doi:10.3390/molecules25020407)
Supplement: Supplementary file 1 [file molecules-25-00407-s001.pdf]

Supplementary Materials: Design, Synthesis and In Vitro Evaluation of the Photoactivatable Prodrug of the PARP Inhibitor Talazoparib

Jiaguo Li [1, 2], Dian Xiao [2], Lianqi Liu [2], Fei Xie [2], Wei Li [2], Wei Sun [1,*], Xiaohong Yang [1,*], and Xinbo Zhou [2,*]

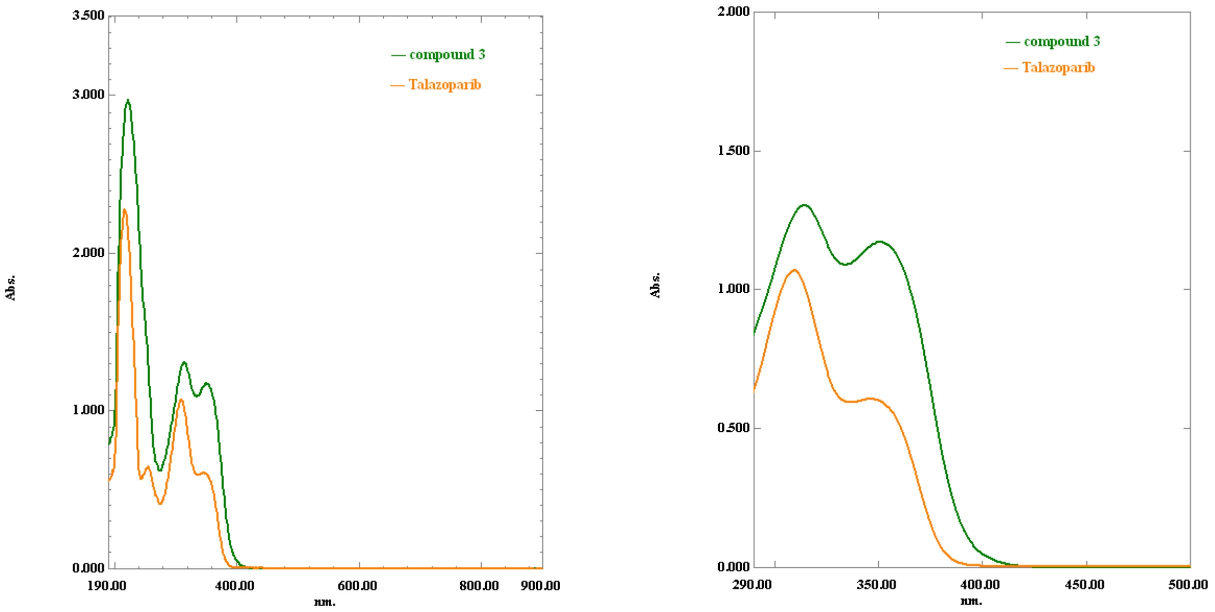

(A) (B)

Figure S1. UV/Vis absorption spectra of Compound 3 and Talazoparib in methanol solution. (A) Full wavelength scanning; (B) Local UV/Vis absorption spectra with a wavelength range of 290-500 nm. Compound 3 and Talazoparib were tested at a same concentration of 0.1 mM. Introduction of the PPG into the parent compound's molecular structure leads to increased light absorption around 365 nm. Wavelengths shorter than 300 nm is highly energetic and can easily damage tissues and cells. Therefore, 365 nm was considered as the optimal wavelength for deprotection.

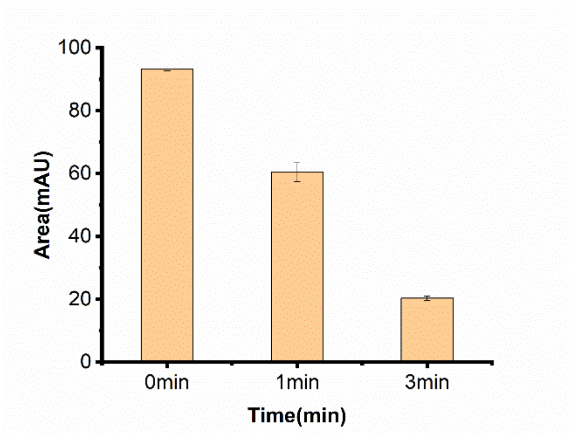

Figure S2. UV-cleavage test on the Compound 3 in culture medium DMEM. Compound 3 were dissolved in DMEM medium containing 0.1 %DMSO and added to 96-well plates at a final concentration of 10 μM. After being irradiated for 1 min and 3 min at 365 nm respectively, the samples were analyzed by HPLC. The peak area is plotted against the irradiation time.

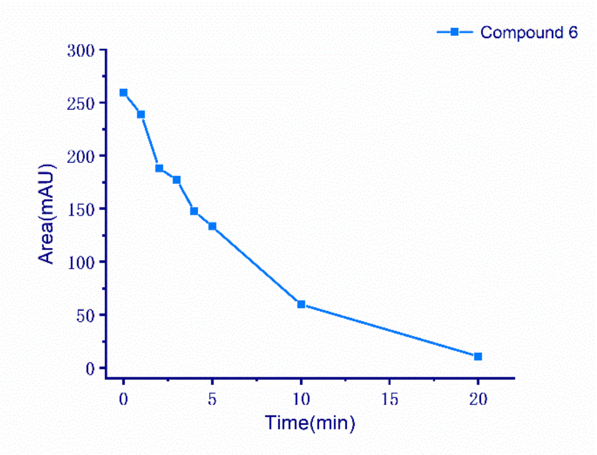

Figure S3. UV-cleavage test on the Compound 6. 20 μM compound in methanol were irradiated at 365 nm for 20 min and analyzed by HPLC. The peak area is plotted against the irradiation time.

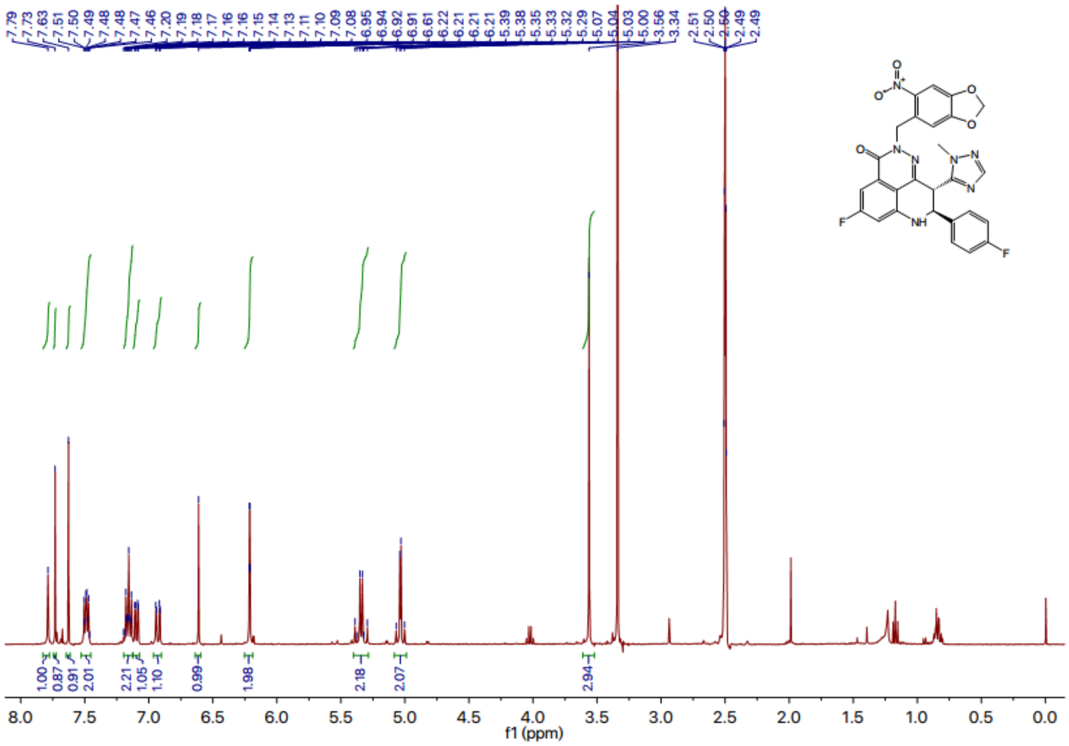

Figure S4. ^{1}H-NMR spectrometry of compound 3.

Qualitative Analysis Report

**Data Filename**	5519.d	**Sample Name**	LJG-0308
**Instrument Name**	TOF G6230A	**Acquired Time**	2019-08-23
**Acq Method**	YCL.M	**Acquired SW**	6200 series TOF/6500 series
**IRM Calibration Status**	**Success**		
**User Chormatograms**			

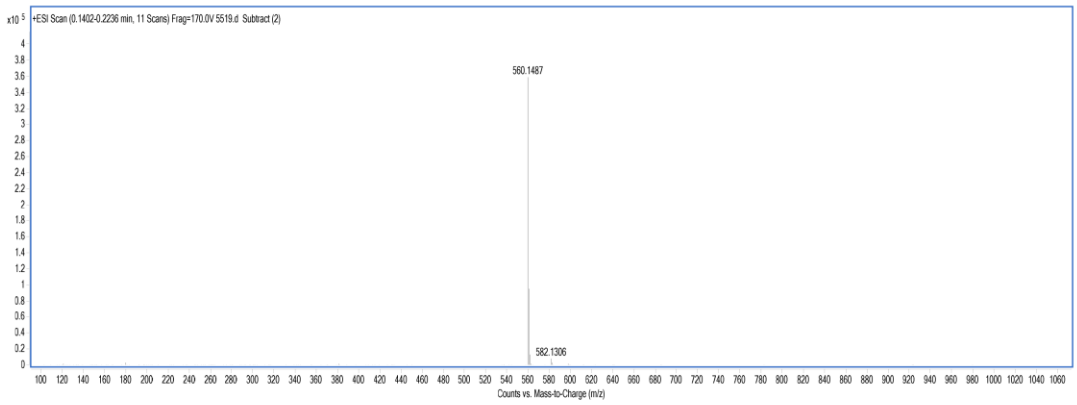

Figure S5. Mass spectrometry of compound 3.

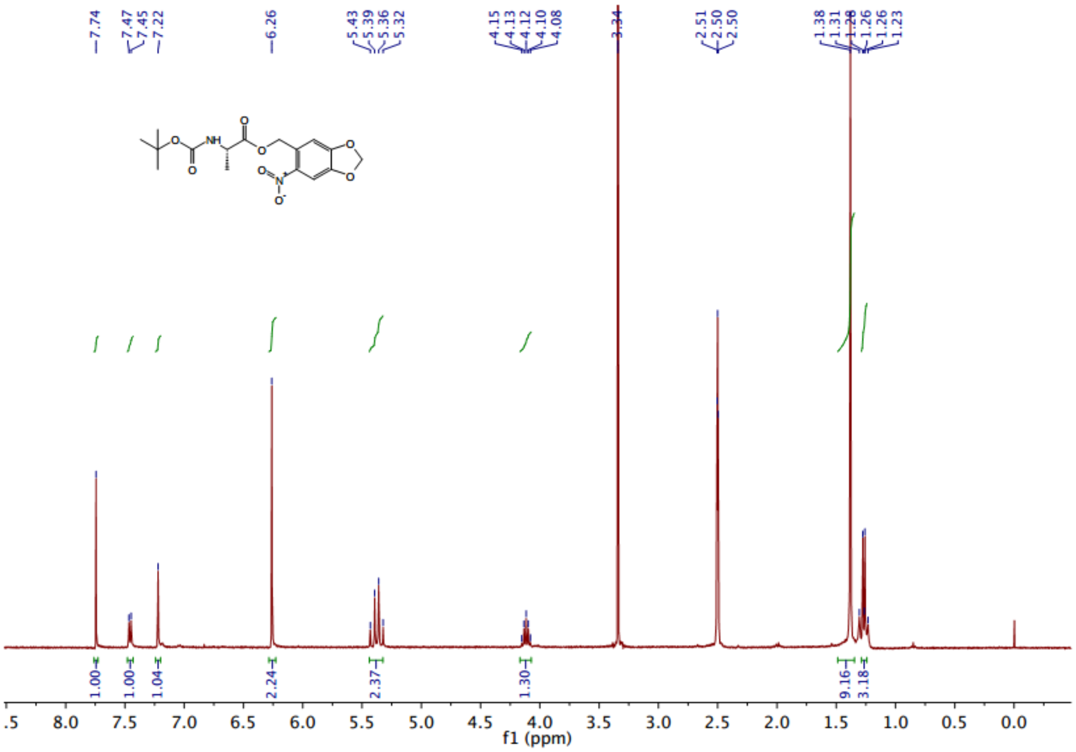

Figure S6. ^{1}H-NMR spectrometry of compound 6.

Qualitative Analysis Report

**Data Filename**	5520.d	**Sample Name**	LJG-0307
**Instrument Name**	TOF G6230A	**Acquired Time**	2019-08-23
**Acq Method**	YCL.M	**Acquired SW**	6200 series TOF/6500 series
**IRM Calibration Status**	**Success**		
**User Chormatograms**			

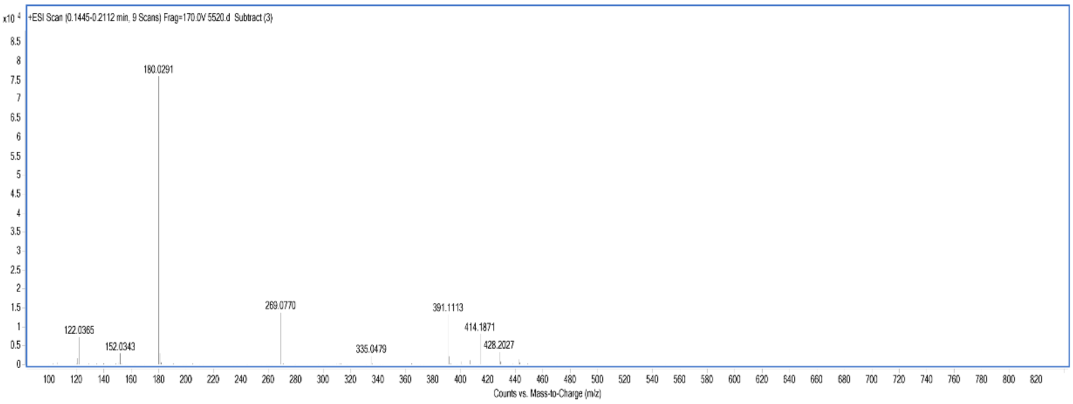

Figure S7. Mass spectrometry of compound 6.
